# Through their eyes’: A qualitative study on the impact of virtual reality on parents’ understanding of visual impairment

**DOI:** 10.1038/s41433-026-04500-6

**Published:** 2026-05-06

**Authors:** Chloe Wagstaff, Michael Williams, Jonathan Jackson, Rebecca McCracken, Sara McCracken, Gerard J. Gormley

**Affiliations:** 1 Centre for Medical Education, Queen’s University Belfast, Belfast, UK; 2 Department of Ophthalmology, Belfast Health and Social Care Trust, Belfast, UK; 3 Angel Eyes NI, Belfast, UK

**Keywords:** Quality of life, Health care

## Abstract

**Background:**

Children born with visual impairment perceive the world differently, which can lead to developmental delays that are often difficult for parents to understand. This uncertainty can leave them unsure of how best to support their child. Virtual Reality (VR) offers simulated experiences and is increasingly being explored as a tool to enhance empathy and understanding - particularly through Point of View (PoV) simulations. PoV VR experiences have the potential to provide personalised insights into a child’s visual impairment (VI). This study addresses the research question: What is the lived experience of parents and carers engaging in a VR simulation replicating their child’s visual condition?

**Methods:**

We conducted a qualitative study to explore the lived experiences of parents and carers using a PoV VR learning experience. Semi-structured interviews, guided by a phenomenological approach, were used to gather in-depth insights. Ten parents/carers of children with VI were recruited and interviewed. Transcripts were analysed using template analysis.

**Results:**

Four themes emerged that captured the depth of participants’ experiences: (1) ‘It all makes sense now’: VR allowing carers to ‘peer’ through their child’s eyes; (2) ‘A flood of emotions’; (3) ‘Seeing into their child’s future’ and (4) ‘Empowerment and advocacy’

**Conclusions:**

PoV VR enhances empathy and understanding of children’s visual abilities, empowering parents and carers to advocate more effectively for their needs. This immersive tool offers deeper insights into the lived experience of VI, underscoring its value in improving support for children with VI and their families.

## Introduction

Globally, a significant proportion of younger people live with visual impairment (VI) and are highly dependent on their parents and carers for additional support [1]. Children and young people with VI face complex challenges associated with their development, further compounded by the difficulty they may have in describing their visual experiences to others [2, 3]. Evidence suggests that parents and carers frequently struggle to gain a true understanding of their child’s visual experiences [4, 5]. There is a strong desire among parents and carers to develop deeper insights into how their child that lives with VI, visually perceives and processes information about the world in which they live [5]. Moreover, parents and carers have voiced concerns about their child’s experiences in educational settings, particularly in enhancing teachers’ understanding of their child’s unique visual abilities, rather than relying on preconceived notions or assumptions [6–9]. Therefore, methods to enhance others’ understanding of how children experience their visual world are required.

Thoreau asked “could *a greater miracle take place than for us to look through each other’s eyes for an instant?”* [10]. By being able to ‘see’ through the ‘eyes’ of others, we might gain a deeper understanding of their lifeworld, challenges, and struggles. To this end, several approaches have been used to provide insight into the visual experiences of individuals with VI. Point-of-view simulation (PoVs) is a type of experiential learning that leverages simulation technologies - such as VR - to immerse an individual in the lived experience of another person. For example, replicating the perspective of someone with VI, enabling users to better understand the challenges and realities faced by that individual. For example, simple spectacles with modified lenses can replicate a range of ophthalmological conditions such as macular degeneration or cataracts [11, 12]. While this form of PoVs has been useful in fostering awareness, it falls short of delivering a fully realistic and nuanced simulated experience. Modified simulation spectacles are typically not adjustable to reflect the unique degree of VI experienced by different individuals [11, 12]. Moreover, users may also be able to look ‘around’ the modified lens to avoid the simulated visual effect [12].

Virtual Reality (VR) can be used to provide individuals with PoV patient experiences. Through the objective technical properties of VR devices, such as frame rate, VR provides ‘immersion’, allowing users to have the subjective experience of being in the virtual world and effectively forgetting they are not in the real one. VR allows the user to be perceptually transported into the body of someone else, an experience known as ‘embodiment’. McLaughlin et al. developed a PoV VR learning experience simulating hearing impairment, which enhanced learners’ empathetic understanding of individuals with hearing loss [13]. PoV VR simulations of two contrasting experiences of being prepped for surgery are described by Hoek et al. [14]. The VR experience was narrative in nature and was found to be useful in creating awareness of therapeutic communication styles amongst anaesthetic trainees. Whilst VR VI simulators exist, there is a paucity of research supporting the use and impact of PoV VR in ophthalmic contexts [15]. A study explored the effect of a documentary shown in VR on losing vision [16]. While participants’ empathy did not significantly increase after the VR experience, the sample size was small, and qualitative findings suggested a shift in perspective regarding VI [16]. Moreover, emerging evidence suggests that VR has the potential to enhance practitioners’ empathic responses toward individuals with VI [17, 18]. However, there is limited evidence on the mechanisms of how PoV VR experiences may support parents and carers in understanding a child’s visual abilities. In examining the phenomenon of PoV VR within an ophthalmological context, it is essential to uncover the nuanced ways in which parents and carers experience and interpret this immersive perspective, particularly regarding its influence on their understanding of their relationship with, and actions toward, their child living with VI. Therefore, in this study we aimed to explore how parents and carers engage with a PoV VR activity and interpret these experiences in relation to their thoughts and behaviours toward their own child with VI. Specifically, we set out to address the following research question: w*hat is the lived experience of parents and carers engaging in a VR simulation designed to replicate their child’s visual condition?*

## Methods

To address our research question, we conducted a qualitative study employing a phenomenological approach to explore participants’ lived experiences of a PoV VR experience of VI. We adhered to the Consolidated Criteria for Reporting Qualitative Research guidelines [19] (See Supplement 1).

### Conceptual orientation

Our study aimed to gain deep, nuanced insights into the lived experiences of parents and carers following their engagement with an immersive PoV VR experience. Phenomenology offers a valuable lens for exploring lived experiences [20, 21]. Rooted in philosophical traditions, phenomenology is increasingly being applied in healthcare qualitative research because of its capacity to shed light on complex phenomena - such as the experience of parents or carers afforded the opportunity to perceive the world through their child’s eyes.

This approach not only attends to conscious experiences but also seeks to uncover tacit (i.e. implicit knowledge that is understood but not formally expressed or easily articulated) and pre-reflective dimensions, assigning meaning through interpretive analysis [22, 23]. Importantly, phenomenology enables researchers to describe and interpret experiences that are complex and emotionally charged, such as those associated with VI. This methodological approach is consistent with the guidance provided by phenomenologists conducting qualitative research [22, 24].

### Ethics

Ethical approval was provided by Queen’s University Belfast (MHLS 22_150). All participants in this study provided written informed consent.

### Setting and context

This study was conducted in collaboration with Angel Eyes NI, a Northern Ireland-based charity that supports families throughout their child’s journey with VI. The organisation provides emotional and practical support to children, families, and carers. One of the tools they use to help individuals gain a deeper understanding of the impact of VI in children is the *Empatheyes* activity, described in detail in the ‘Description of Educational Activity’ section below.

### Recruitment and sampling

An invitation to participate in the study, along with a participant information leaflet, was emailed to parents and carers of children with VI, registered on the Angel Eyes NI database (n = 200). Given that our research focused on the phenomenon of parents’ or carers’ experiences of a PoV VR representation of their child’s VI, the primary characteristic we aimed to explore in depth was their role as being a parent or carer, rather than demographic factors such as gender or age. For this reason, we adopted a convenience sampling method. Convenience sampling can be appropriate within phenomenological research [25–27]. Inclusion criteria for this study include: carers (usually parents) aged 18 years or older who have children with VI not correctable by glasses or contact lenses and who require assistance with daily activities. Carers who had previously used the VR Empatheyes device were excluded. As is typical in phenomenological studies, smaller sample sizes are preferred to allow deeper insight into lived experiences without being overwhelmed by data or promoting a reductionist approach [28]. Therefore, we aimed to recruit up to 8 participants. See Table 1 for details about participants recruited for this study.

**Table 1:** Participant (i.e. parent or carer) details including VI condition of child.

Participant number	Participant pseudonym	Gender as identified by participant	VI condition(s) of child (including Visual Acuity)	VR Software parameters used (including Visual Acuity adjustment)*	Duration of interview (min:sec)
1	Annie	Female	Idiopathic motor nystagmus, moderate photophobia (6/15)	Moderate photophobia; mild contrast sensitivity loss (6/15)	30:33
2	Betty	Female	Bardet-Biedl Syndrome (6/15)	Severe peripheral VF loss; moderate contrast sensitivity loss; moderate photophobia; red/green colour blindness (6/15)	18:54
3	Cara	Female	Albinism, nystagmus (6/24)	Moderate photophobia; mild contrast sensitivity loss (6/24)	22:30
4	Diane	Female	Albinism, mild photophobia (6/24)	Moderate photophobia; mild contrast sensitivity loss (6/24)	22:21
5	Emily	Female	Cerebral visual impairment (6/60-1/60)	Lower VF defect; moderate photophobia; moderate contrast sensitivity loss (6/60-1/60)	16:02
6	Fergal	Male	Congenital retinal folds, optic nerve hypoplasia, hypermetropia (6/12)	Depth perception loss, contrast sensitivity loss (6/12)	20:16
7	Grace	Female	Bilateral foveal hypoplasia, photophobia 6/12 (better eye) and 6/24 (weaker eye)	Mild contrast sensitivity loss; mild/moderate photophobia (6/12) (better eye) and 6/24 (weaker eye)	19:42
8	Helen	Female	Cerebral visual impairment, nystagmus, strabismus (6/15)	Tunnel vision; moderate contrast sensitivity loss (6/15)	17:01

*Software parameters used to adjust the VR visual scenes to match the child’s function, based on known diagnosis and on information in ophthalmic clinic letters (brought by carers), including visual acuity where measured.

### Description of educational activity

**Fig. 1:**
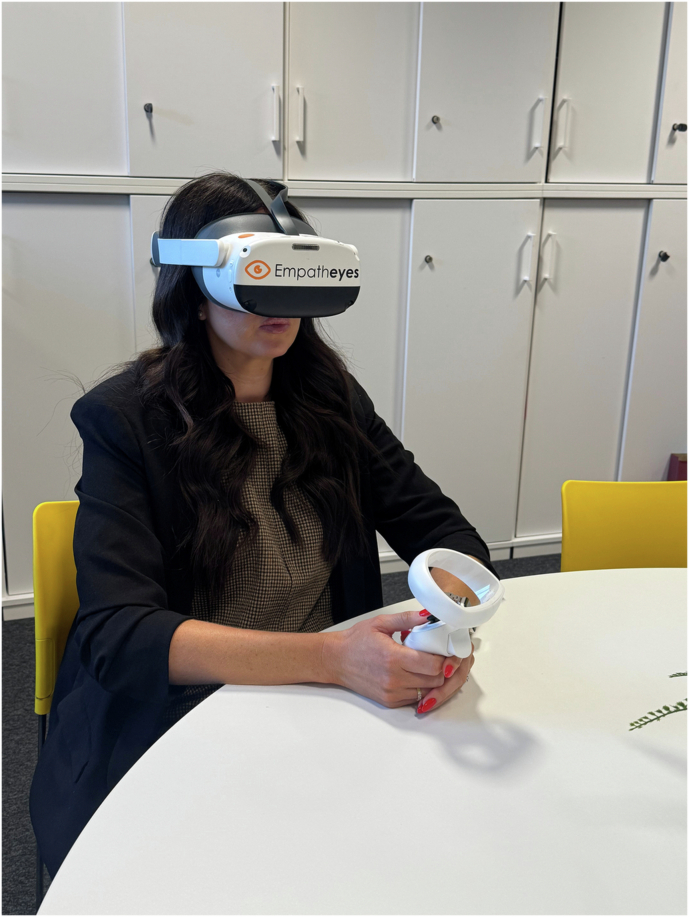
Image of an individual using the Empatheyes VR immersive experience.

We invited participants to engage with a PoV VR simulation called ‘Empatheyes’. Empatheyes is a VR package that simulates a range of VI conditions for the user through a worn headset. It uses eye-tracking, filters that enable the instructor to simulate different types and intensities of VI (visual acuity, contrast sensitivity, visual field size, glare, and colour vision). The degree of VI (mild through to severe) can be modified, and different aspects of VI can be layered on top of each other as desired. Empatheyes enables the user to visualise virtual scenarios (such as a classroom, a living room, a street scene, and a playpark) to recreate for users the experience of VI in a simulated world (See Fig. 1).

**Fig. 2:**
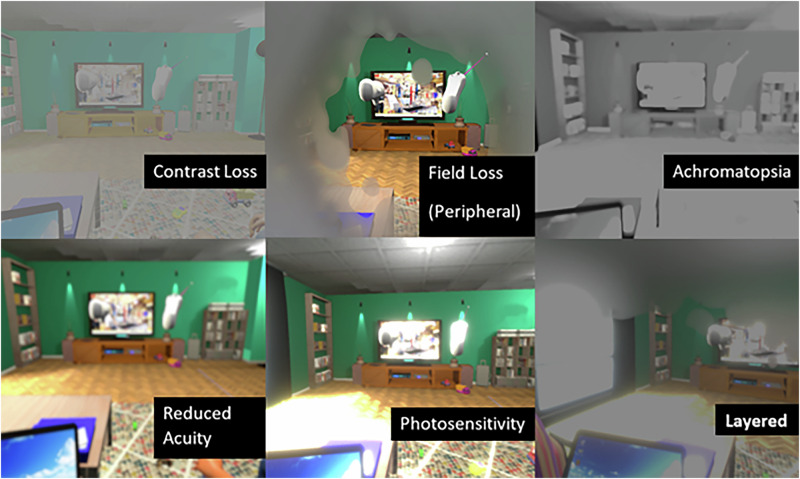
Screen shots of examples of simulations of different aspects of VI using Empatheyes VR experience.

Users can look around the scene by turning their head or body but cannot walk through the scene (See Fig. 2).

Participants were invited to the Angel Eyes (NI) offices in Belfast to take part in the study at different and separate times. Each carer individually experienced the *Empatheyes* PoV VR simulation using a Pico Neo 3 Pro headset. Participants remained seated throughout and were encouraged to explore the virtual environment on one occasion. An *Empatheyes* trainer, present in the room, controlled the VR settings via a mobile application. The experience began with a thoughtful pre-brief and then with a simulation of normal vision, after which the trainer adjusted the visual settings to reflect the participant’s child’s specific diagnosis and level of VI. Participants did not experience other types of VI. The trainer explained the corresponding changes in vision. Information about the child’s VI was provided by carers, who often shared clinic letters to support the customisation. The session was followed by a debrief for participants. Each VR session lasted approximately 40 min. The psychological safety of participants was of paramount importance in this study. In addition to a comprehensive VR simulation pre-brief and debrief, the Angel Eyes NI team contacted participants in the days following the simulation to conduct a well-being check. Furthermore, Angel Eyes NI provides psychological support services that participants could access at any stage. No participant reported a need to use these support services, and no adverse impacts related to the VR experience were observed or reported throughout the study.

### Data generation

**Fig. 3:**
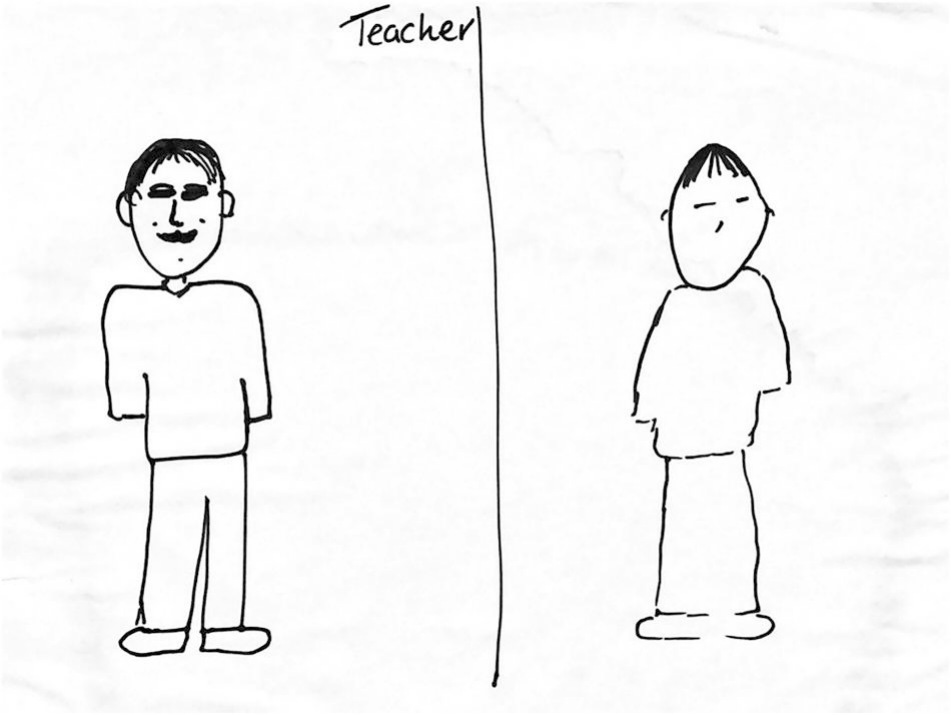
Example of a Rich Picture provided by a participant to help elicit their interview. In this image they depict the loss of the ability to make out facial features. Supplementary file 1 Consolidated criteria for reporting qualitative research (COREQ): a 32-item checklist for interviews and focus groups.

Following the VR experience, a semi-structured interview was conducted by CW with each participant, in addition to taking field notes. CW had no professional relationship with any of the participants. The interviewer received training in qualitative research interviews [29]. An interview guide was used to structure the interviews, but importantly, the interviewer was provided with dialogic space to explore topics that emerged. The interview guide was developed by the research team to facilitate exploration of participants lived experiences. It included key exploratory questions designed to encourage participants to reflect deeply and share their experiences during the interviews. Given the complex and subjective nature of participants’ experiences, we employed an interview elicitation technique to enhance participants’ reflections on the VR activity. *Rich Pictures* is an interview elicitation technique increasingly used in qualitative research [30, 31], and suitable for phenomenological research [32]. By using *Rich Pictures*, participants could convey experiences challenging to verbalise. Following the VR experience, and prior to the qualitative interview, participants were invited to draw an image representing their experience of the VR activity (see Fig. 3 for an example). The interviewer then invited participants to explain their images and used this as a platform to explore their experiences in a more nuanced fashion. Interviews were recorded using a digital Dictaphone. Transcripts were checked for accuracy and anonymised using pseudonyms.

### Data analysis

Interview transcripts of participants’ lived experiences provided the unit of analysis for our study. It is important to note that the *Rich Pictures* were not analysed directly (i.e., they served as an interview elicitation tool). Thematic analysis was conducted using a Template Analysis approach [33]. Thematic analysis is frequently employed in phenomenological research as a means of identifying and organising patterns of meaning within participants’ accounts [34, 35]. In this study, themes serve both as a method for representing participants’ lived experiences and as an interpretive framework for presenting our findings.

This method provided a structured approach to analysing participants’ experiences, researchers’ interpretations, and how the data contributed to a comprehensive understanding of the PoV VR activity. Initially, tentative a priori codes were developed by the research team. These codes were applied to an initial transcript, after which they were refined, excluded, or new codes generated. This process continued with subsequent transcripts until preliminary themes were developed. Through an iterative and reflexive process, the research team developed themes addressing the research question. Once consensus was achieved, all transcripts were coded against the final template. Analysis was concluded when all researchers agreed that a thorough and rich description sufficiently addressed the research question. The research team, consisted of CW (medical student; female), MW (ophthalmologist and academic; male), GG (GP and academic; male), SM (CEO, Angel Eyes NI; female), RM (orthoptist and EmpathEyes trainer; female) and JJ (optometrist and academic; male), maintained reflexivity throughout the study via regular meetings, discussions, and journaling [36]. To ensure the trustworthiness of this study, we followed established qualitative standards of credibility, confirmability, and transferability. Credibility was reinforced through extended engagement with the data, iterative coding cycles, and peer debriefing to verify interpretations. Confirmability was ensured by maintaining reflexivity throughout the research process, critically reflecting on researcher assumptions and potential biases. Transferability was promoted by offering rich, detailed descriptions of the study context and participants’ experiences, allowing readers to evaluate the relevance of findings to other settings.

## Results

Our analysis developed 4 interrelated themes of our interpretation of participants lived experiences of using the immersive PoV VR experience of VI. Participants demographic details, including the condition of their child are outlined in Table 1.

What follows is a detailed description of these themes, and participants quotes to convey these themes and our interpretation.

### ‘It all makes sense now’: VR allowing carers to ‘peer’ through their child’s eyes

Across our dataset, participants universally expressed the meaningful impact the PoV VR activity had on them. We interpret these lived experiences as more than just a novelty, participants conveyed that the VR experience provided a broader worldview - not only of what their child could or could not see, but, more importantly, what this experience might be like from their child’s perspective.

*“It really gives you an insight into what your child is dealing with and what they can see. Until you can see that you honestly don’t know.”* ***(Grace)***

For many participants, we interpreted that this PoV VR activity was more than a visual sensory experience. It evoked a deeper, more embodied understanding of their child’s visual abilities and disabilities. These experiences were emotionally charged, as will be explored in the next theme. Additionally, participants highlighted how the subtleties of what their child could or could not see captivated their attention and elicited strong reactions, as expressed by this participant:

*“As we were watching it, everything was clicking through my head …different scenarios come into your head where you think, that’s why that’s happening”* **(*Cara*)**

From this vantage point, participants were often confronted with the VR experience, which highlighted challenges their child may encounter. It was clear from our interpretation of these experiences that more often than not, subtle experiences that had previously gone unappreciated became more apparent. These frequently centred around key social environments in which the child interacted, such as at home and at school.

*“It has explained a lot of things in the past… difficulties that he’s had that we haven’t understood….it all makes sense now.” (****Betty****)*

### ‘A flood of emotions’

It was clear that participants found the VR activity to be an emotionally charged experience. The emotions they experienced ranged from positive to negative. For example, as described by one participant in terms of positive emotions:

*“I’m glad, so glad that I did it. I would’ve drove anywhere to come and look.” (****Diane****)*

And also negative emotions:

*“I feel a bit down, because of what she has to deal with, what she has to live with.” (****Grace****)*

The emotions evoked by the PoV VR experience varied across participants and within individuals, who could oscillate between positive and negative emotions. Interestingly, despite the emotional state (i.e., positive or negative) evoked by the VR experience, it instilled a deep sense of being able to see what their child could, or could not see, and leverage this emotional stimulus to make a change for their child. For example, when participants experienced positive emotions, they felt this reinforced their commitment to advocate for their child.

*“It has given me more of a sense of, like, empathy with her… I can actually understand why she would be doing those sorts of things…it’s not just her not looking… I’m going to be more patient, definitely.” (****Annie****)*

It was our interpretation that when negative emotions were experienced, participants appeared to harness this emotional stimulus to be activated and willing to make a change. While caregivers often felt that the PoV VR experience was challenging, many were glad to gain a better understanding of their child’s VI and how to better help them in the future.

*“A lot of it is guilt because [child] was only diagnosed in P4, but she’s had this from birth…. I feel bad that it took me that long to pick up on it.”* (***Annie***)

No participants expressed any feelings of distress or reported a negative impact on their well-being or role as a caregiver. Across our dataset, our interpretation was that participants expressed that the experience was worthwhile, despite any negative emotions they might have experienced. As evidenced by this participant:

*“I think it’s very emotional for anybody. I wouldn’t say that it’s in any way easy to watch. I think I did find it uncomfortable at times, but I know that it’s for the best to see it. It’s all very positive to be able to understand it.”* (***Cara***)

Finally, the intensity of caregivers’ emotions seemed to be influenced by how long their child had been living with a VI. Caregivers of children with a more recent diagnosis often exhibited stronger emotional responses, likely due to their ongoing adjustment to their child’s condition.

### Seeing into their child’s future

Across our dataset, participants experienced a profound sense of reflection on their child’s future. Our interpretation of this PoV VR immersion prompted them to imagine the challenges their child might face as they grow older. These reflections often centred on potential day-to-day life challenges, such as educational opportunities, future careers, and relationships. This interpretation is exemplified by this participant

*“When he was diagnosed, I was told that he would never drive a car, and now I understand. I was in denial… there’s no way he could drive a car.”* (***Diane***)

For many participants, the PoV VR experience reinforced their preconceived worries about their child’s future. However, there were moments when it also encouraged them to consider challenges and life opportunities they had not previously contemplated. One participant’s account illustrates this shift:

*“You start questioning, you know, what he will have to deal with in his life, I suppose. School and then from there. He keeps going on about wanting to be a fireman, and obviously he’s not going to be a fireman if his eyesight is as bad as that.”* (***Fergal***)

From this vantage point, it was clear from our interpretation that participants gained deeper empathy not only for the challenges their child currently faces but also for the potential obstacles they may encounter in the future. As one participant reflected:

*“It provided such a better insight, and it made me appreciate more of the difficulties, you know, from her perspective.”* (***Helen***)

While such realisations were challenging for participants, they were often viewed as a catalyst for advocating more effectively for their child.

### Empowerment and advocacy

Overwhelmingly, the immersive PoV VR experience created a sense of empowerment in participants to advocate for their child. It was evident from our interpretation and analysis that experiencing the world through the eyes of a child with VI instilled a profound sense of injustice and a strong desire to take action. As typified by this participants account:

*“maybe I’m fighting this battle and I shouldn’t be fighting, but seeing it? No, I’m going to keep fighting …… if that’s what she is seeing then I’m going to keep fighting for her.”* (***Annie***)

Participants often related their desired actions to the specific context of their own child. For instance, they considered how changes could be made in their day-to-day activities, particularly within the home environment. These reflections highlighted how the immersive experience motivated participants to implement practical, child-centred changes in their immediate surroundings, emphasising the personal and actionable nature of their advocacy.

*“It would make me think longer about what I could do to help her…Like spacing out her bedroom, putting things more organised… how you make things more accessible.”* (***Grace***)

A dominant experience shared by participants was their increased desire to advocate for their child at school as a result of the PoV VR experience. Many expressed a newfound awareness of the challenges their child faced in an educational setting, particularly regarding accessibility and support. The immersive nature of the VR simulation allowed them to see firsthand the barriers children with VI encounter in the classroom. We interpreted this as the impact being not just to heighten empathy, but also to motivate participants to take action - through direct communication with school staff, advocating for changes in the classroom environment, or seeking additional resources to better support their child’s learning. This interpretation is supported by this quote:

*“I feel that I would be better equipped if I was going into school to speak to the SENCO [special educational needs co-ordinator] or the teachers involved. I have a lot more knowledge now behind me to be able to go and explain to them how his vision is affected in the classroom, rather than what it would’ve been beforehand” (****Betty***)

Given the profound impact the PoV VR experience had on them, participants also expressed a strong desire for others to have the same immersive experience. We interpretate this motivation was driven not only by the positive influence it had on their own perspectives but also by the belief that it could be a powerful tool for advocating for children with VI. Participants recognised the potential for the PoV VR experience to foster greater empathy and understanding, which could ultimately lead to meaningful changes in both attitudes and behaviours. For example, one participant shared how they hoped the experience would be used in schools and community settings to raise awareness and prompt action to better support children facing similar challenges.

*“If a teacher … had sat with that headset on and had that experience… the lightbulb would come on and they would realise the difficulties that he does have.” (****Betty***)

## Discussion

In this study, we aimed to explore and interpretate the lived experiences of parents and carers engaging in a PoV VR simulation designed to replicate their child’s VI condition. Our findings indicate that such a PoV VR immersion can provide parents and carers with an empathic (i.e. the ability to understand and share another person’s feelings, thoughts, or experiences) and empowering (i.e. providing the confidence or ability to take control of their own decisions or actions) experience. Naturally, they have a close connection with their child and their visual abilities. However, the PoV VR experience offered a new layer of understanding of their child’s view of the world and provided a more nuanced insight into their struggles. Although VR could never truly replicate an individual’s VI experiences, our findings support the notion that PoV VR can offer a meaningful and reflective experience, enhancing parents’ and carers’ understanding of their child’s visual abilities and how these impact their social world.

Previous research indicates that parents often face challenges in understanding medical jargon [37]. Ophthalmic data, e.g. visual acuity, is often conveyed in numerical form which can be difficult for a parent to fully appreciate. However, as evidenced by the findings from our study, PoV VR simulation can provide a unique stimulus to help parents and carers translate such terminology into a more meaningful and embodied experience, as one of our participants explained, *“could be understood by anyone.”* It was evident that the PoV VR experience nurtured a renewed empathic response in the parents and carers of children with VI. Not only empathy with their child but also empathy with their future, affirming their desire to effect change to provide inclusive conditions for them to lead their life to the fullest potential. Evidence suggests that developing empathetic responses in parents can drive child attachment security and be beneficial in maintaining close parent-child relationships [38].

Prior research would suggest that PoVs can be an emotive experience [13]. Our research findings on PoV VR depicting a child’s VI are no different. Although many of the emotions were positive, some of the emotions evoked in participants were more negative, such as guilt and frustration. It must be noted that at no time during our study did a parent or carer feel distress or anxious, even in the days after the VR experience with the follow up ‘check in’ with participants. Instead, such emotional responses provided a deep-seated sense of empowerment to advocate (i.e. to actively support or argue in favour of something – such at their child rights and life chances) for their child and were perceived as an experience for the better. The experience was empowering and evoked a sense of a force for good. Participants expressed gladness in gaining a new perspective on their child’s VI and using this experience to support their child as they navigate the world and their future. These findings echo previous research exploring the impact of PoVs in other conditions such as hearing impairment [13].

Given the importance of education in their child’s development and the significant time spent in schools, parents and carers had a renewed focus on advocating for their child in educational settings. Participants expressed that teachers and professionals involved with their child’s education often have limited understanding of VI and how to make necessary changes in the classroom, an issue which has previously been raised in other studies [6–9]. Research indicates that parents of children with disabilities agree that teachers often lack adequate training on how to manage students with disabilities, with a review of education of students with VI referencing teachers’ feelings of unpreparedness [8, 9].

### Strengths and limitations

Our study is the first to explore the experiences of parents and carers using a PoV VR experience designed to replicate their child’s visual condition. Despite our promising results, findings must be considered within the limitations of this study. Firstly, findings from phenomenological studies are meant to be more transferable rather than generalisable. Transferability in qualitative research refers to the extent to which findings can be applied or adapted to other contexts or settings. This is achieved by providing rich, detailed descriptions (“thick description”) of the research context and participants, enabling readers to judge whether the results are relevant to their own situation rather than being generalisable. Other research methodologies would be required to provide generalisable findings. Despite the range of VI conditions represented in this study, they may not be generalisable to other VI conditions. Lastly, the longer-term impact of the PoV VR experience on parents and carers was beyond the scope of this study.

### Implications for practice and future research

Our study highlights the potential of PoV VR experiences to enhance parents’ and carers’ understanding of their child’s VI, fostering empathy and empowerment. This suggests that integrating PoV VR simulations into support programs for families of children with VI could be beneficial. Whilst further research is required, our study provides an important impetus to expand this work and explore whether immersive PoV VR experiences can meaningfully enhance understanding, empathy, and advocacy among parents and carers of children living with VI. Future research should explore the long-term effects of PoV VR experiences on parents and carers, including how these influence advocacy efforts and support strategies over time. Additionally, studies could investigate the effectiveness of PoV VR simulations across a broader range of VI conditions and in different cultural contexts to determine their generalisability. Research could also examine the impact of VR experiences on other stakeholders, such as teachers and healthcare professionals, to understand how VR can improve their understanding and support for children with VI.

## Conclusion

Caring for a child with VI presents unique challenges for parents and carers. Our study suggests that a PoV VR experience can offer a powerful learning opportunity, enhancing their insights, empathy, and understanding of the child’s visual abilities. By immersing parents and carers in a simulated visual world, PoV VR provides a deeper, more nuanced perspective on the child’s experiences and struggles. This understanding appears to empower them to advocate more effectively for their child’s rights and needs, ensuring access to the best possible support for thriving. The potential of PoV VR to foster empathy and advocacy highlights its value as a tool for improving the lives of children with VI and their families, and warrants further research in this area.

## Summary

### What was known before

- Children born with visual impairment experience the world in fundamentally different ways, which can contribute to developmental delays that are often challenging for parents to interpret and understand.
- This uncertainty can leave parents feeling unsure about how best to support their child’s unique needs.
- Virtual Reality offers promising potential to deliver personalised insights into a child’s visual impairment, helping caregivers better understand their lived experience.

### What this study adds

- Virtual Reality can enhance empathy and understanding of children’s visual abilities, empowering parents and carers to advocate more effectively for their needs.
- VR immersive technology provides deeper insights into the lived experience of visual impairment (VI), highlighting its value in improving support for children with VI and their families.

## Supplementary information

Supplemental File 1

## Data Availability

The data that support the findings of this study are available from Queen’s University Belfast (QUB), but restrictions apply to the availability of these data, which were used under licence and ethical approval for this current study and so are not publicly available. The data are, however, available from the authors upon reasonable request and with the permission of QUB.
